# Angel and devil: the protective immunity and pathogenic inflammation of tissue resident memory T cells in ulcerative colitis

**DOI:** 10.3389/fimmu.2025.1518339

**Published:** 2025-03-07

**Authors:** Xintong Wang, Jiaqi Zhang, Lihui Fang, Xudong Tang

**Affiliations:** ^1^ Institute of Digestive Diseases, Xiyuan Hospital of China Academy of Chinese Medical Sciences, Beijing, China; ^2^ Graduate School, China Academy of Chinese Medical Sciences, Beijing, China; ^3^ Department of Gastroenterology, Xiyuan Hospital of China Academy of Chinese Medical Sciences, Beijing, China; ^4^ Graduate School, Beijing University of Chinese Medicine, Beijing, China

**Keywords:** tissue-resident memory T cells, ulcerative colitis, inflammatory bowel disease, recurrence, immune

## Abstract

Ulcerative colitis (UC) is an incurable autoimmune disease. Patients with UC endure the burden of recurrent flare-ups and face a substantial economic burden due to long-term medication. The complex etiology and unclear pathogenesis pose a significant challenge to the development of effective and curative treatments. Recent research indicates that local memory at the site of inflammatory intestinal mucosa in UC is closely associated with the persistent presence of tissue-resident memory T (TRM) cells. TRM cells, a subset of memory T cells, exhibit long-lived, low-migration characteristics. These cells reside in tissues, where they provide immediate immune protection while also contributing to chronic, localized inflammation. The presence of TRM cells in the inflamed intestinal mucosa of UC patients is a crucial factor in the recurrence of the disease. However, the process involved in the formation and differentiation of TRM cells within the intestinal mucosa remains poorly understood. Various surface markers, transcriptional networks, and signaling pathways regulate the formation and maintenance of TRM cells in the intestine. To further understand the role of TRM cells in UC pathogenesis, we have summarized the latest findings to pave the way for the development of future targeted therapies.

## 1 Introduction

Ulcerative colitis (UC) is a chronic, recurrent and currently incurable inflammatory bowel disease (IBD) characterized by uncontrolled inflammation, which leads to damage to the bowel (1). Over the past decade, there has been a notable increase in the global prevalence of UC, particularly in developing countries and regions such as Asia and Eastern Europe (2, 3). Patients with UC experience a significantly reduced quality of life due to associated symptoms, including diarrhea or bloody stools, abdominal pain, and fecal urgency. Additionally, they bear a substantial physical, psychological and economic burden due to disease recurrence and the elevated risk of cancerization (4).

The treatment goals for UC are focused on achieving rapid endoscopic remission or combined endoscopic and histologic remission, collectively referred to as mucosal healing. In addition to conventional treatments, although significant advances in biological therapeutic strategies such as anti-tumor necrosis factor-α (TNF-α) drugs, anti-leukocyte adhesion molecule preparations, kinase inhibitors, interleukin (IL) -12 and IL-23 antagonists (5–9), maintaining clinical remission and preventing recurrence remains a substantial challenge for most UC patients, even after drug withdrawal. The recurrence of UC is often characterized by periodic remission and exacerbation, which can occur spontaneously or in response to drug treatment. A meta-analysis revealed that the overall risk of UC recurrence after discontinuing anti-TNF treatment is 38% (10). Apparently, current therapeutic measures remain insufficient to fully address these complexities (11). Therefore, it is imperative to gain a comprehensive understanding of the mechanisms underlying the recurrence and to develop effective prevention strategies.

Tissue-resident memory T (TRM) cells, a subtype of memory T cells, have been identified as a critical cell type in the immune system. Under physiological conditions, only 5%~10% of effector T cells could escape from apoptosis after clearing pathogens or antigens (12). These cells have the potential to transform into TRM cells through the induction of transforming growth factor-β (TGF-β) or IL-15 (13). Following this transforming, TRM cells are stably established in barrier organs or tissues, including the skin, gut, and respiratory mucosa. These cells provide rapid immune responses to reinfection without being activated by antigen-presenting cells (14, 15). However, growing evidence indicates that TRM cells may also target autoantigens or persistently exposed antigens, contributing to autoimmune diseases such as autoimmune hepatitis, rheumatoid arthritis and UC (16–18).

Current evidence indicates that TRM cells may play a central role in the mechanisms of intestinal inflammation and local recurrence in UC. The potent proinflammatory and tissue-resident properties of TRM cells may be responsible for the recurrent episodes and specific inflammation localization in UC patients (19, 20). Therefore, a detailed discussion of the cellular phenotype of TRM cells, as well as the underlying immunomodulatory and inflammation mechanisms is necessary.

## 2 TRM cells in intestinal mucosa

### 2.1 Cell surface markers

Although without exact and consistent findings, various cell markers have been observed on TRM cells. We have summarized some of the key cell surface markers in the intestine based on recent studies.

**Figure 1:**
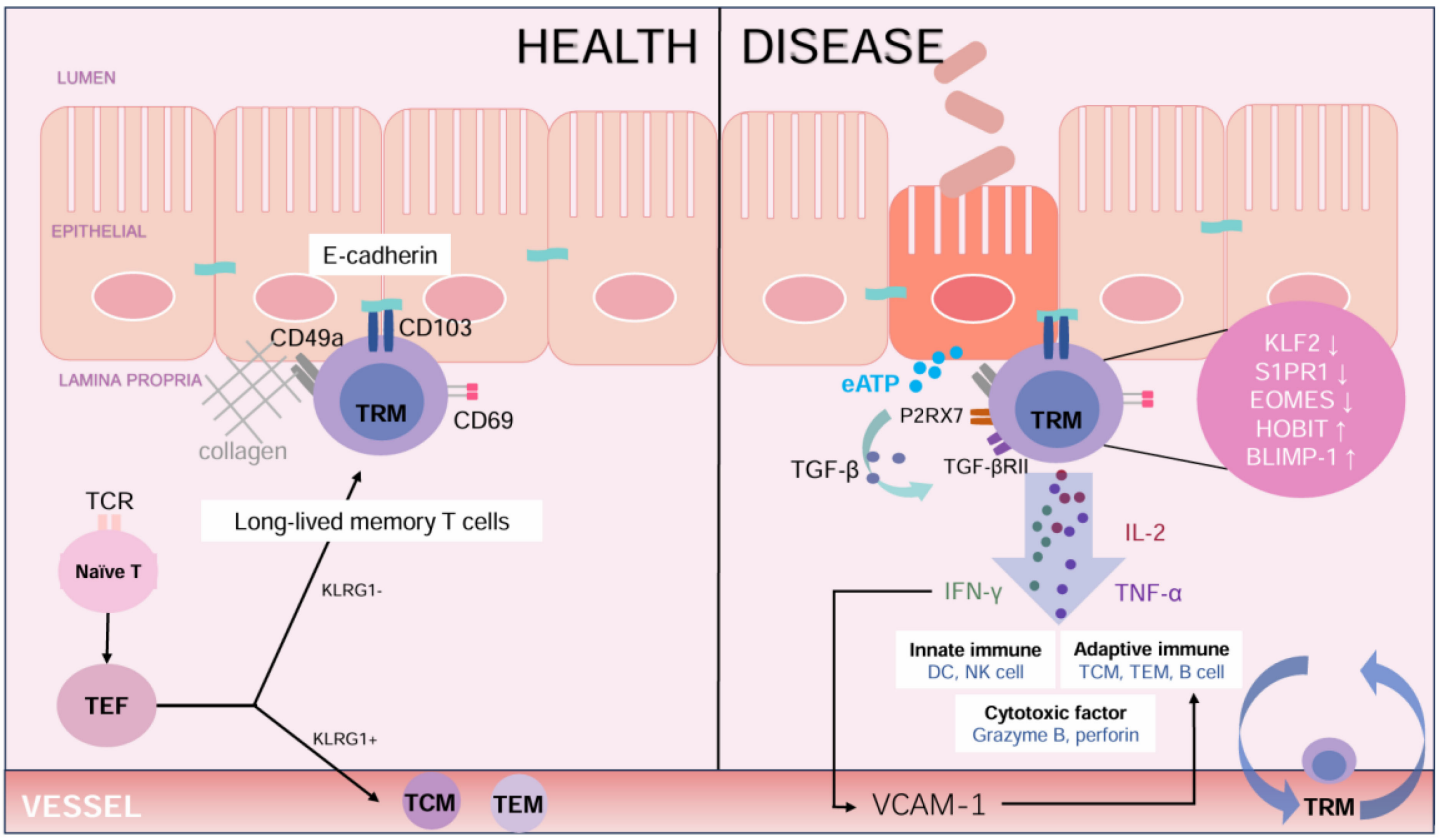
The role of TRM cells in maintaining intestinal health and contributing to disease. In healthy colon, TRM cells primarily express CD103 and CD69 to maintain local immunity and homeostasis. The binding of Ecadherin supports the retention of these cells, while cytokines such as TGF-β promote the development of CD103^+^TRM cells. In Ulcerative Colitis, TRM cells exhibit a proinflammatory phenotype, characterized by secreting IL-2, IFN-γ, and TNF-α. These cytokines activate both innate and adaptive immune responses and release cytotoxic factors like granzyme B and perforin. Molecular changes, such as decreased expression of KLF2 and increased expression of BLIMP-1 expression, enhance the inflammation response. Signals from extracellular ATP (eATP) and P2RX7 receptor exacerbate immune activation, contributing to tissue damage in UC. (TCR, T cell receptor; TEF, effector T cell; TCM, central memory T cell; TEM, effector memory T cell; TRM, tissue resident memory T cell; KLRG1, Killer cell Lectin-like Receptor G1; eATP, extracellular Adenosine Triphosphate; P2RX7, Purinergic Receptor P2X Ligand Gated Ion Channel 7; TGF-β, Transforming Growth Factor-β; TGF-βRII, Transforming Growth Factor-beta Receptor II; IL-2, Interleukin-2; IFN-γ, Interferon-γ; TNF-α, tumor necrosis factor-α; KLF2, Krüppel-like Factor 2; S1PR1, Sphingosine-1-phosphate Receptor 1; EOMES, Eomesodermin; HOBIT, Homeobox Protein Hox-11-Like; BLIMP-1, B Lymphocyte Induced Maturation Protein 1; VCAM-1, vascular cell adhesion molecule-1.).

CD103 and CD69 were defined as classic phenotypic markers on TRM cells (21) (**Figure 1**). CD103 (αE integrin), a receptor for E-Cadherin, is predominantly expressed on intestinal CD8^+^ TRM cells, where it promotes the residency of TRM cells (22). It is considered to be a CD8^+^ TRM-residency-specific molecule (23), as most CD4^+^ T cells do not express CD103 (24). In normal barrier tissues, TGF-β signaling promotes CD103 expression, ensuring the long-term maintenance of CD103^+^ CD8^+^ TRM cells in nonlymphoid barrier tissues (25). During intestinal inflammation, the expression of CD103 can be influenced by cytokines such as interferon (IFN) -β and IL-12. These inflammatory mediators may modulate TGF-β-mediated CD103 expression, potentially inhibiting or altering its induction in different cellular contexts (26). Since E-cadherin is primarily expressed in the intestinal epithelium, it may have limited impact on the lamina propria where CD103^+^ cells locate (27).

CD69 serves as a marker of early T cell activation and is closely associated with T cell residency (28). During the development of TRM cells, TGF-β negatively regulates Forkhead Box Protein O1 (FoxOl) through the PI3K-Akt pathway, downregulating Kruppel-like factor 2 (KLF2) (29). KLF2 is a transcription factor critical for T cell trafficking, which directly downregulates sphingosine-1 phosphate receptor 1 (S1PR1) and indirectly upregulates CD69, preventing T cells egress from the intestine (30). The lack of S1PR1 is also a method to identify TRM cells. Compared with CD103, CD69 is a secondary marker, as CD69 is not an essential residency marker in the intestine of mice (31). However, in humans, CD69 is instrumental in distinguishing between TRM cells and circulating memory T cells (32).

Only 25% of TRM cells in the colon express both CD103 and CD69, TRM cells lacking CD103 or CD69 can also be found (33). Their generation depends on the location and whether they arise from local tissue infection (34), as these cells play a rapid response role in secondary infections (35). Compared to CD103^+^ TRM cells, intestinal CD103^-^ TRM cells have been demonstrated to possess greater effector functions, including the ability to produce higher levels of granzyme A (36). CD103^-^ TRM cells share similar transcriptional profiles with circulating T cells, rapidly producing IFN-γ, TNF-α, and IL-2 while retaining memory of infection within tissues after clearance (37, 38). The cytokine IFN-γ, largely regulated by signal transducer and activator of transcription 4 (STAT4), is a key driver of CD103^-^TRM cell differentiation (39). Some CD8^+^ CD103^-^ TRM cells also express high levels of β2-integrin (40), while others express Killer Cell Lectin-like Receptor G1 (KLRG1), another receptor for E-Cadherin, which can compete with CD103 (41). Intestinal CD103^-^ TRM within the intraepithelial layer are derived from KLRG1^+^T cells (42). These KLRG1^+^ T cells exhibit enhanced survival and developmental plasticity, enabling the generation of CD103^-^ TRM (42).

Furthermore, CD49a, the α chain of integrin α1β1, is an immunomodulatory and adhesive molecule that interacts with type IV collagen in the lamina propria to establish tissue residency, promoting the accumulation of CD8^+^ TRM cells (43, 44) (**Figure 1**). CD49a expression is upregulated in CD69^+^ TRM cells, contributing to the adhesion between T cells and intestinal epithelial cells (45). This is critical for the retention of TRM cells in the gut.

CD161, also called Killer Cell Lectin-Like Receptor C1 (KLRC1), is a C-type lectin-like receptor expressed by adult CD8^+^ T cells (46). It is a subtype of CD8^+^ T cells that produces significant amounts of cytotoxic meditators, including granzyme B and perforin. Additionally, these cells express high levels of the transcription factors Eomesodermin (EOMES) and T-box expressed in T cells (T-bet) (47), which predominantly regulate the expression of cytotoxicity mediators and cytokine production (48). Intermediate levels of CD161 are highly expressed in the colon. In inflamed tissues of IBD patients, CD161 is enriched in CD103^+^ TRM cells, and the majority of CD161 in the colon co-expresses CD69 (47). CD161^int^ CD8^+^T cells provide rapid immune protection against pathogens within the gut.

CD39 and CD73 are two regulatory markers expressed by TRM cells, involved in nucleotide-metabolizing that regulate immunity and inflammation (49). After the release of the inflammatory signal adenosine triphosphate (ATP), CD39 converts ATP to adenosine monophosphate (AMP), which is subsequently dephosphorylated by CD73 to produce adenosine (50). These enzymes facilitate the transition from ATP-driven, proinflammatory immune cell activity an adenosine-mediated, anti-inflammatory states. As these markers are typically expressed at high levels on regulatory T cells (51), reduced levels of CD39 with IBD may indicate a compromised regulatory function. Studies have shown that CD39 and CD73 are highly expressed on CD103^+^ CD8^+^ TRM cells, suggesting that these cells possess an immunosuppressive function (51). Human TRM cells expressing CD39 and CD73 may contribute to the maintenance of intestinal immune homeostasis.

Although CD69 and CD103 are considered the most prominent surface markers of TRM cells, they are not specific to TRM cells. Currently, the study of cell surface markers is not comprehensive, other potential markers of intestinal TRM cells include CD44 and CD101 (40). In particular, the specific TRM subsets in UC patients need to be further refined and studied.

### 2.2 Differentiation of intestinal TRM cells

Multiple metabolic pathways are involved in the generation and function of TRM cells. At present, a greater number of studies have been conducted on TRM cells in the small intestine than in the colon. Most of the findings were derived from experimental lymphocytic choriomeningitis virus (LCMV) mouse infection and clinical research in patients.

Purinergic Receptor P2X, Ligand Gated Ion Channel 7 (P2RX7) has recently been identified as a key signaling molecule associated with the formation of TRM cells. Given the abundant antigenic substances and microbiota in the colon, extracellular adenosine triphosphate (eATP) from the colon microbiota activates the P2RX7 (52), which enhances the sensitivity of TGF-β in CD8^+^ TRM cells (53). TGF-β has been proven to contribute to the development of TRM cell characteristics and the establishment of transcriptional networks (54). In the intestine, naive CD8^+^T cells are activated and differentiated in response to antigenic stimulation. TGF-β in the intestine environment accelerates the apoptosis of short-lived effector cells while promoting the rapid formation of memory precursor cells (55). This process occurs through the simultaneous downregulation of KLF2 and SIPR1, along with the upregulation of CD69 and CD103 (56) (**Figure 1**). The signaling molecule Smad3 also contributes to CD103 regulation (57).

Additionally, P2RX7 is an ion channel that responds not only to eATP but also to Nicotinamide Adenine Dinucleotide (NAD^+^), playing a role in cell death via the ART2.2/P2RX7 pathway (58). Given its dual function, the role of P2RX7 in the development and function of TRM cells warrants further investigation. CD38 is an enzyme expressed on T cells that exerts regulatory effects on both the metabolism of NAD^+^ (59) and the development of TRM cells. CD38 serves as a marker distinguishing TRM cells from circulating memory T cells across various organs. Deletion of CD38 has been observed to impact TGF-β-dependent CD103^+^ TRM cell subpopulations in epithelial tissues (60).

Newly published research (61) has revealed that depletion of nutrient-dependent lysosomal signaling nodes activates the transcription factor EB (Tfeb), and deficiency in Follicular Lymphoma Overexpressed Gene (Flcn) enhances protective responses in intestinal TRM cells, with the Flcn-Tfeb axis mechanistically inhibiting retinoic acid-induced CCR9 expression to promote TGF-β-mediated lineage differentiation programs. Subsequently, the expression of CCR9 contributes to the differentiation of intestinal CD103^+^ TRM cells via retinoic acid signaling in mesenteric lymph nodes (62).

Although many new pathways are being discovered, studies on colonic TRM cells remain limited compared to those on small intestine TRM cells. Most of the mechanism-related studies have been conducted in mice and require further validation due to the heterogeneity of TRM cells.

### 2.3 Transcriptional factors of intestinal TRM cells

The development, maintenance, migration, and function of intestinal TRM cells are regulated by a complex network of transcription factors. The transcriptional factors Blimp-1/Hobit and Eomes/T-bet play crucial but distinct roles in this process.

Blimp-1 and Hobit are transcription factors involved in TRM cell generation and regulation (63), encoded by Zfp683 and PRDM1 (64), respectively. They exhibit a high degree of overlap in their genomic binding sites, homologous sequences, and synergistic action that collectively drive T cell lineage differentiation (65). Approximately 30% of the genes related to tissue-resident functions in TRM cells are regulated by Blimp-1 and Hobit (64). Blimp-1 and Hobit suppress the expression of Klf2, S1pr1, T-cell transcription factor (Tcf), and C-C motif chemokine receptor 7 (Ccr7), thereby contributing to the residency of TRM cells in tissues. Ccr7 is a critical regulator of lymphocyte egress from peripheral tissues, which is usually absent in TRM cells (64). The core transcription factor Runx3 could induce chromatin accessibility and regulate Blimp-1 (66, 67). In CD8^+^T cells, Blimp-1 knockout alone is insufficient to induce granzyme B expression (68). However, Hobit is responsible for maintaining granzyme B expression, and in conjunction with Blimp-1, sustains the cytotoxic function of TRM cells (69). Following antigen rechallenge, TRM cells may experience a decrease in Hobit expression, leading to the formation of circulating memory cells (70). The development and maintenance of intestinal CD8^+^ TRM cells are impaired in mice with a double knockout Blimp-1 and Hobit, resulting in alleviated colitis (19). In a mouse model of colitis, the Hobit^-^ Blimp-1^-^ CD4^+^ TRM cells lead to reduced chemokines expression, impaired leukocyte recruitment, and decreased expression of proinflammatory molecules (71).

The T-box transcription factors Eomes and T-bet are involved in the complex regulatory mechanisms that control TRM cell differentiation and maintenance in the colon. Eomes is a key transcription factor to the differentiation and effector function of CD8^+^ T cells (72), inducing the production of proinflammatory substances such as IFN-γ, granzyme B, and perforin (73). Compared to the small intestine, the number and function of colonic TRM cells do not depend on EOMES (33). This phenomenon may be attributed to the distinct microenvironment present in the small intestine and colon. Ectopic expression of Eomes in a mouse model enhances the pathological properties of CD8^+^ T cells, supporting that Eomes promote inflammatory and pathological states in CD8^+^ TRM cells (73, 74). The coordinated downregulation of Eomes and T-bet promotes the generation of CD8^+^ CD103^+^ TRM cells and enhances their responsiveness to TGF-β signaling (75). A recent study (76) discovered that depletion of T-bet, a key molecule along with Hypermethylated in cancer 1 (Hic1) in regulating intestinal TRM cell differentiation, partially restored Hic1 expression and significantly rescued the formation of intestinal CD103^+^ TRM cells in the absence of TGF-β receptor signaling.

## 3 TRM cells in UC

The powerful proinflammatory properties and abnormal activation of TRM cells in the colon may explain the pathogenesis of UC flare-ups. In the colon, pathogens in the intestinal lumen are captured by antigen-presenting cells, such as macrophages and dendritic cells in the mucosal layer, which subsequently activate naïve T cells. These naïve T cells differentiate into effector T cells and memory T cell subsets, which migrate to sites of intestinal inflammation in response to cell adhesion molecules, including chemokine receptors. Among these subsets, memory T cells can be generated during the early stages of the immune response and at various stages of differentiation. In the later phase of the inflammatory response, some memory T cells return via the bloodstream, while others are retained locally in the intestinal epithelium or lamina propria. Upon encountering the same antigen, CD4^+^ and CD8^+^ TRM cells are rapidly activated, proliferate, and generate cytotoxic responses. If this immune response is not properly regulated, extensive immune cell crosstalk occurs, leading to intestinal mucosal damage and chronic intestinal inflammation.

In this way, TRM cells in UC are primarily categorized into CD4^+^ and CD8^+^ subsets, which are consistent with the general classification of T cells. CD8^+^ TRM cells are mainly located in the intestinal epithelium, but they can also be found in the lamina propria, where CD4^+^ TRM cells mainly exist (77). It was found in IBD patients that, CD4^+^ TRM cells produce more IL-17 than circulating CD4^+^ T cells (78). IL-17 is an important proinflammatory cytokine that can induce a variety of cells, such as epithelial cells, fibroblasts and endothelial cells, to produce proinflammatory cytokines and chemokines, such as IL-6, IL-8, TNF-α, etc., thereby aggravating intestinal inflammation. The number of CD4^+^ TRM cells in the lamina propria of colonic mucosa of active UC patients is significantly higher than that of healthy people and is closely related to the frequency of clinical recurrence of UC (19). So CD4^+^ TRM cells mainly play a proinflammatory role in UC, depletion of these cells in mice with transfer colitis resulted in significant alleviation of colitis flare-ups.

The research of CD8^+^ TRM cells in UC remain limited. A study on healthy participants showed that CD103^+^ CD8^+^ TRM cells in the gut directly kill infected or damaged cells through cytotoxic mechanisms involving granzyme B and perforin, and they mainly express IFN-γ, IL-2, and TNF-α upon activation (51). Single-cell sequencing analysis revealed significant heterogeneity in T-cell subsets between healthy controls and UC patients. In UC patients, Eomes-mediated pathologic CD8^+^ TRM cells were expanded and the KLRG1^+^EOMES^+^ITGB2^+^ subpopulation was enriched (74). The target genes of Eomes, including IFN-γ, granzyme A and KLRG1, displayed enhanced inflammatory and cytolytic characteristics (74). In the inflamed mucosa of CD patients, there was a relative decrease in the number of CD103^+^CD8^+^ TRM cells and an increase in the number of KLRG1^+^CD8^+^TRM cells (79). One subset of these cells exhibited a stronger response to T-cell receptor stimulation, while the other subset demonstrated enhanced cytotoxic potential, proliferative capacity and expression of T helper cell 17 genes (79). A clinical study analyzed that in both CD4^+^ and CD8^+^ TRM cells, the proportion of CD103^+^ TRM cells in IBD is reduced during the inflammation phase, indicating that CD103^+^ TRM cells may be involved in maintaining mucosal homeostasis and regulating the immune response (80, 81). Earlier experiments in mice showed that CD103^+^ CD8^+^ T cells could alleviate transfer ileitis and inhibit the proliferation of CD4^+^ T cells through TGF-β (82). Immunomodulatory markers such as CD39 and CD73 are mainly expressed on the surface of some CD8^+^ TRM cells (82). Thus, CD8^+^ CD103^+^ TRM cells may support homeostasis in noninflamed gut. Additionally, the expression of CD69+TRM cells mRNA and proinflammatory cytokines, including IFN-γ, IL-13, IL-17A and TNF-α, has been found to be elevated during the active phase of the disease (19). Therefore, it is worthwhile to investigate the potential of CD103^+^ TRM cells in maintaining intestinal immune homeostasis.

Emerging evidence demonstrated that TRM cells present a new potential target for the treatment of recurrent UC (71). CD8^+^ TRM cells, activated by IFN-γ, have been implicated in immune checkpoint inhibitor-associated colitis and have been shown to respond to JAK inhibitors, making them a promising target for it (83). New therapies targeting T cell trafficking, including the α4β7 antagonist peptide PTG-100 (84), anti-β7 integrin antibody Etrolizumab (85), the α4-integrin antagonist AJM300 (86), and sphingosine-1-phosphate (S1P) receptor modulator Ozanimod (87), are closely related to the residency and recirculation of TRM cells. Etrolizumab, which selectively targets α4β7 and αEβ7 integrins, is designed to impede the intestinal homing of inflammatory T cells. This consequently helps their residency in the intestinal mucosa, including the surface molecule CD103 (88). In a phase 3 randomized controlled trial, Etrolizumab demonstrated superior efficacy and safety compared to adalimumab and placebo in patients with moderately to severely active UC (89). Ozanimod, an S1P receptor modulator, has been shown to prevent lymphocyte mobilization to inflammatory sites. Furthermore, the drug has been found to be more effective than a placebo in improving endoscopic and mucosal healing in patients with moderately to severely active UC (90). The P2X7 receptor antagonist AZD9056 induced clinical remission in patients with moderate-to-severe CD, but failed to result in a notable reduction in serum C-reactive protein and fecal calprotectin (91). The calmodulin phosphatase pathway plays a pivotal role in P2RX7 signaling, which is implicated in the generation of TRM cells (92). Inhibitors of this pathway are employed in the induction therapy for acute severe UC, despite their dual function (92). This outcome presents a challenge for future clinical research on the P2RX7 receptor. In summary, the identification of pathogenic TRM cells represents a promising opportunity for the development of potential therapeutic strategies for the management of IBD.

In conclusion, TRM cells play a crucial role in both innate and adaptive immune systems by providing local immune defense in the intestine under normal physiological conditions. In pathological states, abnormally activated TRM cells have been observed to trigger severe intestinal inflammation and localized inflammatory flare-ups. The advent of single-cell transcriptomics, T-cell receptor library analysis and mass spectrometry technology provides the possibility to further elucidate the pathophysiological mechanisms of TRM cells at the cellular level. In light of these findings, we summarize the role of different phenotypes of TRM cells in the development of UC, with a particular focus on UC recurrence. We also provided an overview of the regulatory factors involved in the formation, differentiation, residence, migratory and recycling of TRM cells, and identified the potential of specific targets for the treatment of UC recurrence. In sum, it can be proposed that similar to the two sides of a coin, different subgroups of intestinal TRM cells exhibit both protective and pathogenic functions. The amplification and migration of abnormal TRM cells are the key targets in UC recurrence and are involved in triggering systemic symptoms. However, limitations remain. The inconsistency in results between human and animal studies on TRM cells is attributed to the tissue specificity of TRM cells and differences in the clinical backgrounds of patients. To identify the optimal balance between the protective and pathogenic functions of TRM cells, extensive studies are necessary to uncover the detailed characteristics and functions of TRM cell subpopulations, which is essential for the development of effective UC therapies.
